# Influencing factors of users’ shift to buying expensive virtual gifts in live streaming: Empirical evidence from China

**DOI:** 10.3389/fpsyg.2022.997651

**Published:** 2022-11-30

**Authors:** Junwei Cao, Guihua Zhang, Dong Liu, Meng Shang

**Affiliations:** ^1^Department of Business, Yangzhou University, Yangzhou, China; ^2^Department of Business, Yeungnam University, Gyeongsan, Republic of Korea; ^3^Department of Global Business, Yeungnam University, Gyeongsan, Republic of Korea; ^4^School of Flight, Anyang Institute of Technology, Anyang, Henan, China

**Keywords:** switch intention, information overload, social presence theory, live streaming, consumption intention

## Abstract

The market size of live streaming on the Internet, in which the streamer earns profit by prompting users to give virtual gifts through emotional labor, is getting bigger and bigger. However, most users will only buy cheap virtual gifts in live streaming, therefore exploring how to promote users to buy expensive virtual gifts is a valuable topic in live commerce research. Based on social presence theory and information overload theory, this study used the PLS-SEM method to investigate the factors influencing live streaming users to shift from buying cheap virtual gifts to buying expensive virtual gifts, and analyzed the moderating role of information overload in these relationships. The results show that immediate interaction anxiety, verbal intimacy, and virtual physical intimacy positively influence users’ shift to purchasing expensive virtual gifts, and that perceived network size and perceived financial risk are negative factors in users’ shift to purchasing expensive virtual gifts. Information overload has a moderating role in the relationship between immediate interaction anxiety and switch intention, and it also plays a moderating role in the relationship between perceived network size and perceived financial risk on users’ switch intention.

## Introduction

Live streaming refers to the service of real-time delivery of content on a live streaming platform on the Internet by streamers based on the Internet, using live tools such as smart phones or computers (Zhou et al., 2019). With high interactivity, strong immersion, high entertainment value and high user stickiness, live streaming has developed into an online entertainment program with a certain scale (Zhou et al., 2019).

Virtual gift consumption is a major profit source for live streaming platforms, which constantly promote users to purchase virtual gifts by increasing visual stimulation and adding gamification elements, etc. (Zhang et al., 2019). Most virtual gifts range in value from 0.1 CNY to 2,000 CNY (about USD 0.014~USD 285), and once a user gives a streamer a virtual gift, special effects and gift notifications will appear in the live stream (Zhou et al., 2019). The more expensive the virtual gifts given by users, the more they will be recognized by the streamer, and the higher the intimacy they may achieve with the streamer (Zhou et al., 2019).

However, the current *per capita* spending in live streaming platforms is low, and in particular, the number of expensive virtual gifts purchased is much lower than the number of cheap virtual gifts (Zhu et al., 2017). Generally, virtual gifts from users on live streaming platforms are considered expensive when the value of the gift reaches USD 14.2 or more (Zhang et al., 2019). A survey showed that 20% of users on live streaming platforms bought 97% of virtual gifts (Tu et al., 2018), of which 57.3% spent only USD 1–10 on virtual gifts to the streamer, and only 2.9% of users gave more than USD 50 in virtual gifts to the streamer (Lee et al., 2019). Therefore, promoting users to shift from buying cheap virtual gifts to expensive virtual gifts is an important issue for the future development of the online live streaming industry, it is also an important topic that must be addressed in live commerce research. However, there are gaps in the current research in this area. Therefore, the first research question of this study was the following:

*RQ1*: Which factors influence live streaming users to shift from buying cheap virtual gifts to buying expensive virtual gifts?

In live streaming, the streamer creates an atmosphere from the utilitarian value and emotional value perspectives that repeatedly emphasizes the excellent performance and price advantage of the product, reminds consumers of the product’s limited time and quantity, and constantly encourages consumers to make a purchase decision (Wongkitrungrueng and Assarut, 2020). At the same time, the streamer also repeatedly calls consumers by intimate titles (such as family, darling), in order to establish a close virtual relationship, so that consumers do not see the streamer as the provider of the product, but rather as a family member or friend they can trust (Park and Lin, 2020). These actions may cause user information overload. Information overload has been shown to confuse consumers when making decisions (Chen et al., 2009) and affect their purchase decisions, especially in online shopping (Furner and Zinko, 2016). Therefore, does information overload in live streaming have an impact on users’ switch intention to buy expensive virtual gifts? What is the mechanism of such an influence? Therefore, the second research question of this study was the following:

*RQ2*: What is the role of information overload in the shift of live streaming users from buying cheap virtual gifts to buying expensive virtual gifts?

## Literature review and theoretical background

### Virtual gift purchase behavior

The essence of virtual gifts on live streaming platforms is to monetize live content. Monetization of live content helps monetize users’ emotions toward the streamer through virtual gifts (Johnson and Woodcock, 2019). Users use real money to buy digital currency provided by the live streaming platform, which can be used to buy virtual gifts (Wohn et al., 2018). Virtual gifts are a derivative of chat messages in online live streaming and often have gamification elements (Siutila, 2018), such as special effects that occur when gifts are given. Meanwhile, users who give expensive virtual gifts are treated with respect. For example, the live channel lists the information of the most expensive gift senders and welcomes them when they enter the channel, which makes them honorable in the eyes of both the streamer and other users (Zhu et al., 2017), and gains them recognition from the streamer (Wohn et al., 2018). The main studies on user engagement and purchase intention in live streaming are shown in Table 1.

**Table 1:** Virtual gift purchase behavior.

Source	Sample	Theory base	Methods	Key findings
Hu et al. (2017)	412 Chinese respondents	Social identity theory	Survey	The group identification of user-hosts and users is positively correlated with their continuous viewing intentions. Personal experience determines the user’s identification with the streamer. Engagement, cognitive communication, and empathic contagion influence the user’s identification with the group. The content of the live stream (genre type) moderates the effect of these two identities on continuous viewing.
Hilvert-Bruce et al. (2018)	227 Twitch users	Use and gratification theory	Survey	Social interaction, sense of community, meeting new people, entertainment, seeking information and lack of real-life external support promote user engagement in live streaming. Users are more likely to watch live streams with fewer than 500 users.
Lee et al. (2019)	239 Korea respondents	-	Survey	The perceived value of immediate sponsorship, affection for the creator and vulnerability induced by the creator have a positive impact on the user’s intention to continue sponsoring.
Zhou et al. (2019)	Douyu.com	Social interaction and arousal theory	crawler	Audience-to-audience interactions can influence the arousal level of the audience through stimuli extracted from Danmaku, which prompt gift-giving. The types of stimuli associated with Danmaku are the presence of others, social competition, and emotional stimuli.
Hu and Chaudhry (2020)	327 Chinese respondents	S-O-R	Survey	Economic ties (price advantages, such as giving points or discounts), social ties and structural ties (added value, such as streamers trying on clothes or style recommendations) influence consumers’ engagement and their emotional commitment (attachment) to the streamer and the live streaming platform. Economic ties do not directly influence consumer engagement, but need to be mediated by emotional commitment.
Liu et al. (2020)	214 Chinese respondents	Social presence theory	Survey	The visual scene positively influences the user’s perceived enjoyment, the communication function and the social presence. Social presence has a positive effect on perceived enjoyment. Perceived enjoyment has a positive impact on the user’s intention to adopt.
Hou et al. (2020)	210 Chinese respondents	Uses and gratification theory	Interviews and surveys	The appeal of sex and humor, the display of social status and interactivity play a considerable role in user behavior in live streaming, and the impact varies in different types of live marketing.
Li and Peng (2021)	403 Chinese respondents	Attachment and flow theories	Survey	Live streamer characteristics (credibility and attractiveness) can stimulate users’ emotional attachment to the live streamer and thus promote their gift-giving intention. Live scene characteristics (telepresence and entertainment) can stimulate users’ flow experience in live streaming. Users’ flow experience can influence their emotional attachment to the live streamer.

When monetizing users’ recognition as virtual gifts, it is possible to purposefully guide users’ purchasing behavior. First, the emotional relationship between the user and the streamer directly predicts the user’s purchasing behavior (Hilvert-Bruce et al., 2018). Live streaming is a kind of social relationship-based marketing that is skilled at monetizing users’ emotions (Wohn et al., 2018), and users’ emotions toward streamers are an important promotion factor to buy virtual gifts (Lee et al., 2019). Live streaming platforms focus on maintaining social ties between users and streamers, building virtual emotions and interactions between users and streamers based on social ties, establishing a sense of identity for the streamer, and prompting users to generate emotional commitment to the streamer and influence virtual gift buying behavior (Hu and Chaudhry, 2020). By creating various intimate situations, the live streaming platform constantly emphasizes the significance of virtual emotional relationships to users in order to raise their emotional awareness and make them buy virtual gifts (Wang, 2019). At the same time, streamers also stimulate users by increasing their own attractiveness, improving entertainment feelings, etc., triggering users’ emotional commitment and flow experience; the stronger the stimulation, the more users tend to buy virtual gifts (Zhou et al., 2019; Hu and Chaudhry, 2020; Li and Peng, 2021). Users’ emotions toward the streamer reduce their vulnerability to requests from the streamer and increase their willingness to give gifts (Lee et al., 2019). These relationship-and emotion-based stimuli not only come from the live streaming platform or the streamer, but also partly from other users, as reported by a study that combined social interaction theory with text mining techniques to analyze the text chat content in live streaming. This study found that the presence of others and social competition increased users’ perception of the streamer’s emotional distance and promoted the purchase and giving of virtual gifts (Zhou et al., 2019).

The creation of visual scenes and instant communication functions in live streaming promote the interaction between users and streamers, thus influencing users’ sense of social presence; eventually, the sense of hedonism is developed by the social presence, allowing users to accept live streaming technology (Liu et al., 2020). User–streamer interactions have been shown to have a positive impact on user purchase behavior in various types of live streaming (Hilvert-Bruce et al., 2018; Li and Peng, 2021). A study that investigated user engagement on Twitch noted that social interactions are a motivating behavior in live streaming, significantly predicting user sentiment generation, viewing, subscribing and purchasing behavior, and that these interactions are more likely to occur on smaller live channels, which can facilitate meaningful social interactions (Hilvert-Bruce et al., 2018). By purchasing virtual gifts, users increase their social interaction with streamers, and streamers tend to show closer emotional distance to users who purchase virtual gifts (Zhou et al., 2019), which highlights the social status of users in the live streaming platform; social status has been confirmed to have a positive impact on live streaming users’ consumption intentions (Hou et al., 2020). Finally, interaction can also trigger purchase behavior by stimulating users’ sense of identity. A study based on social identity theory points out that the parasocial interaction and cognitive exchange between the user and the streamer cause the user to identify with the streamer and continue to watch, and eventually generate purchase behavior (Hu et al., 2017).

In addition to emotional factors of purchase intention in live streaming platforms, there are numerous studies suggesting diverse influencing factors from various aspects. A research study on college students found that users’ need for recognition and the perceived value of virtual gifts from streamers promoted users’ spending on live streaming platforms, while perceived subjective norms, perceived payment inconvenience, and perceived financial burden hindered users’ willingness to spend (Lee et al., 2019). Two other studies point to a relationship between the personal characteristics of the streamer and the user’s willingness to purchase: for instance, the streamer’s style of dress and sense of humor both positively influence the user’s willingness to purchase on live streaming platforms (Hou et al., 2020; Hu and Chaudhry, 2020).

In general, the analysis of users’ willingness to buy virtual gifts for streamers on live streaming platforms has begun to be increasingly comprehensive, and many recent studies have designed various algorithms based on these earlier studies and developed various new technologies to predict and guide users’ purchasing behavior on live streaming platforms (Andronie et al., 2021; Pelau et al., 2021; Hopkins, 2022; Nica et al., 2022; Kliestik et al., 2022a,b). However, almost all current studies have ignored the analysis of users’ willingness to purchase expensive virtual gifts. Therefore, it is meaningful to clarify the influences of users’ willingness to buy expensive virtual gifts on live streaming platforms.

### Social presence theory

Social presence theory is a concept in the field of telecommunications research, and is a model for analyzing social psychology in media communication from the perspective of social cues (Parker et al., 1978; Gunawardena, 1995). Social presence has two levels of meaning: the first is the significant degree of interaction with others, and the second is the degree of change in interpersonal relationships as a result of the interaction (Parker et al., 1978).

Social presence theory defines the degree of physical proximity induced through the use of various media. This physical proximity is considered to be a sense of social presence induced through media, i.e., the virtual interaction of body and emotions with others through media. Social presence theory consists of two main concepts: immediacy and intimacy. Based on these two concepts, two sub-concepts can be extended: efficiency and non-verbal communication (Parker et al., 1978).

Immediacy in social presence theory refers to the perceived power given to users in media platforms. This ability allows users to perceive the urgency and importance of interactions, such as impatience to view and respond to messages (Wiener and Mehrabian, 1968). There are two important factors involved in immediacy: the responsiveness of the media platform and the response of the other party after receiving the interaction. When discussing these two factors, it must be considered how to measure the responsiveness of both, which gives rise to the concept of efficiency (Smedlund and Faghankhani, 2015). Efficiency highlights the need to provide users with perceived efficiency at the media platform level and at the interaction level.

Intimacy was originally defined as the degree to which two people develop a sense of closeness and belonging to each other (Zelizer, 2000), and intimacy is often thought to be influenced by physical distance, conversation topics, and nonverbal communication. Combined with social presence theory, intimacy can be thought of as the closeness that arises through media interactions (Argyle and Dean, 1965), and intimacy reflects the degree to which users perceive closeness or interact with exclusive partners in digital platforms, whether face-to-face or online; this perception is influenced not only by verbal communication but also by nonverbal behaviors such as physical distance, eye contact, smiling, and body language, i.e., the concept of nonverbal communication is derived (Parker et al., 1978; Dixson et al., 2016).

In recent years, more and more research has focused on the role of social presence theory in the operation of online platforms. Early studies suggest that users are inclined to purchase more goods if the e-commerce platform provides them with a sense of social presence (Weisberg et al., 2011). Social presence can be divided into cognitive presence and affective presence, both of which are conducive to promoting users’ recognition of social networking sites (Chang and Hsu, 2016). Social presence also enhances users’ sense of belonging and entertainment on social platforms (Gao et al., 2017), and these ultimately influence users’ trust in social commerce platforms and their willingness to purchase (Hajli et al., 2017). A study analyzing the effect of social presence on users’ intention to purchase virtual items in online games found that social presence has a positive effect on this intention (Jin et al., 2017). Another study analyzing individuals’ intention to participate in social commerce on online platforms reported that social presence has a significant effect on this intention (Hassan et al., 2018). Social presence also enhances the celebrity effect in social commerce marketing. Studies have shown that, on Instagram, consumers feel a stronger sense of social presence when they are exposed to posts from celebrity-endorsed brands, making the products of these brands more trustworthy (Jin et al., 2019).

Social presence is also an important motivation for users to watch live streaming (Ham and Lee, 2020). The visual scene and communication function in live streaming affect the user’s social presence, and the hedonic sense triggered by social presence makes the user finally accept live streaming (Liu et al., 2020). These interactive functions help streamers and users communicate effectively, and moderate the relationship between users (Lin, 2021), create a sense of screen presence and emotions(Guo et al., 2019), guide users into the state of streaming experience, and increase users’ willingness to buy virtual gifts (Li et al., 2018). They also promote users’ sustainable purchases on live streaming platforms (Clement et al., 2020).

There is a strong link between social presence and the maintenance of online user relationships (Nadeem et al., 2020), and social commerce marketing will be more effective if the dimensions of social presence are effectively utilized in eliciting consumers’ purchase intentions (Jiang et al., 2019). For example, in live streaming, users give virtual gifts to the streamer as a way to gain recognition (Johnson and Woodcock, 2019). The most direct expression of recognition is the intimacy given by the streamer after gifts are given to them. For example, streamers repeatedly call consumers by intimate titles (e.g., family, darling) to establish intimate virtual relationships (Park and Lin, 2020), and in addition to intimate titles, the presentation of some physical gestures can also promote users’ identification with the streamer and facilitate the generation of interactive intimacy (Jodén and Strandell, 2021). In addition, some studies even point out that gender and sexuality are also key factors in online live streaming marketing (Ruberg and Brewer, 2022), and that these help to build intimacy. Ultimately, the sense of online intimacy felt by users generates a stream of emotions that triggers the act of giving virtual gifts (Wang, 2019). In summary, it is appropriate for this study to use social presence theory to analyze the antecedents of users’ shift to purchasing expensive virtual gifts.

### Information overload in marketing

Information overload is defined as when the current demand for information processing is higher than the actual ability to process information. The criterion to determine whether the information processing demand matches the processing capacity is time, i.e., whether the capacity to process information within a certain time frame matches the amount of information that needs to be processed (Eppler and Mengis, 2004). Information overload generally occurs during the retrieval, analysis and decision-making processes of information (Eppler and Mengis, 2004). When individuals are affected by information overload, it will be difficult for them to effectively synthesize information, which will trigger anxiety and feelings of stress, ultimately leading to dysfunction and poor decision making (Cao et al., 2021).

The impact of information overload on consumer purchase intentions in the online environment has received widespread attention. Chen et al. (2009) suggested that the advantage of e-retailers is that they can deliver a wealth of information to customers, but that the information overload caused by too much information leaves consumers in a confused state when making decisions. One researcher conducted an experiment with consumers by building eight different shopping websites, and the results of the experiment pointed out that more product information increases consumer trust, which reduces consumer price awareness and leads to consumer purchase intentions (Huang et al., 2013). Meanwhile, the results of a survey of 1,396 online shoppers in Spain confirmed that information overload can positively influence consumers’ willingness to purchase online. The survey also confirmed that information overload can also increase consumers’ perceived risk and reduce their willingness to purchase. The researchers of this study also stated that their findings add some controversy to the research on the relationship between information overload and customer purchase intentions (Soto-Acosta et al., 2014). Another study showed that information overload reduces consumer trust and purchase intentions, especially in mobile web shopping (Furner and Zinko, 2016).

As a social platform, users inevitably encounter information overload when watching live streaming. Because of the various profit-increasing programs developed on live streaming platforms (Zhang et al., 2019), which put pressure on streamers, streamers are encouraged to deliver information more frequently on buying virtual gifts to users. In addition to directly requesting users to buy virtual gifts, streamers also frequently indirectly prompt users to buy virtual gifts, such as by using a lot of words and sounds that increase users’ virtual intimacy, showing more of their good looks and sexy bodies, and sometimes even showing immoral content such as sexual teasing (Lee et al., 2019). It has been confirmed that displaying good looks, sexy bodies and even immoral content are widely used strategies by streamers in live streaming for income (Lee et al., 2019; Hou et al., 2020), and that nonverbal actions containing sexual advances are also increasing in live streaming (Zolides, 2020); although most platforms have regulatory measures for sexually provocative actions, the measures are imperfect and many vulnerabilities still exist (Ruberg, 2020). A large amount of highly stimulating information will continuously stimulate users through their visual and auditory senses, and a large amount of suggestive purchase information, especially non-verbal information, will significantly increase the burden of users in processing information. When this exceeds the information processing capacity of users, they may enter a state of information overload.

## Research model and hypothesis

### Research model

**Figure 1:**
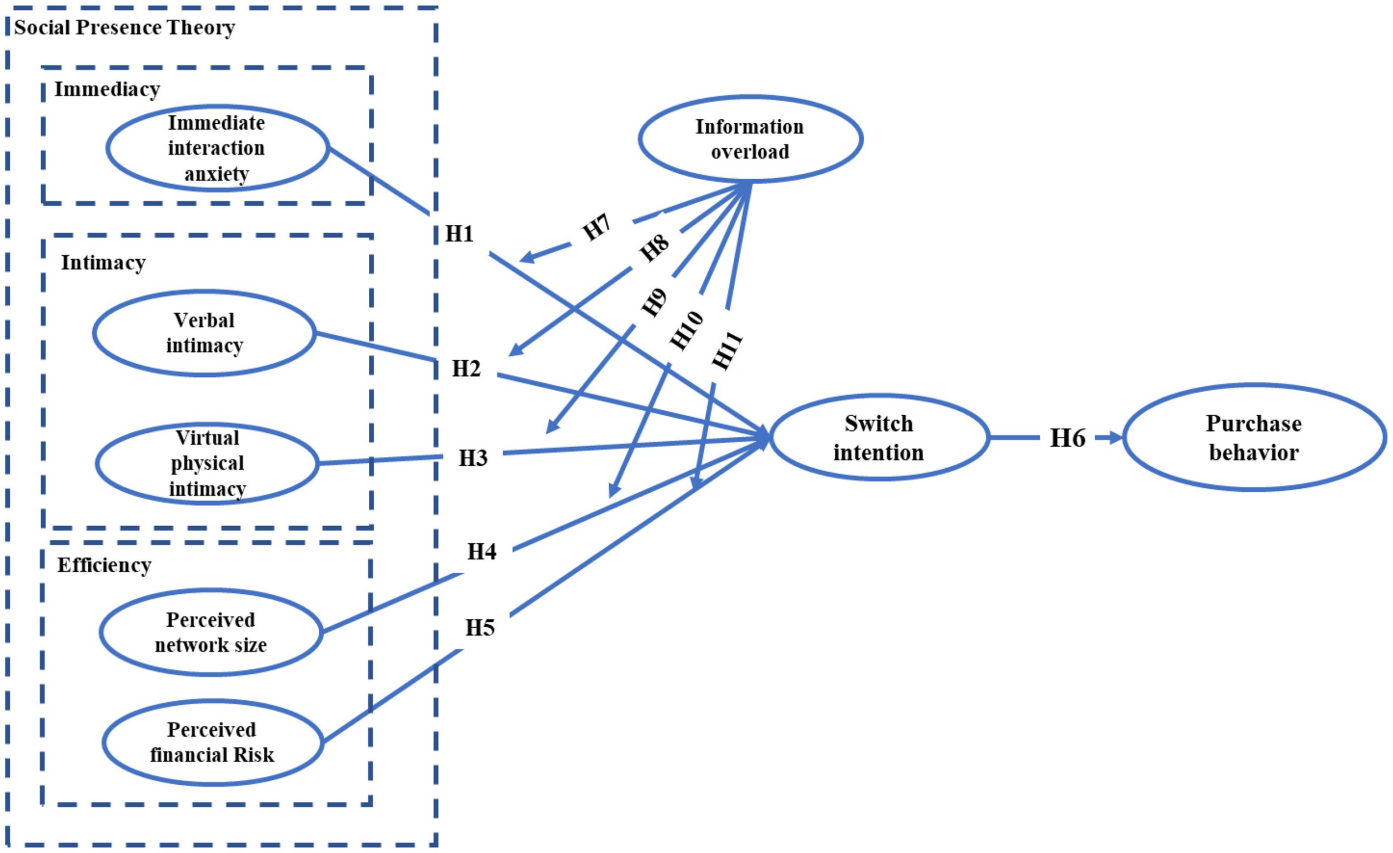
Research model.

Based on social presence theory, this study proposes a model to investigate the factors that influence live streaming users to switch from buying cheap virtual gifts to buying expensive virtual gifts. Based on the main concepts in social presence theory, combined with the literature on the consumption intentions of live streaming users, we identified three factors that facilitate users’ shift to purchasing expensive virtual gifts: immediate interaction anxiety, verbal intimacy, and virtual physical intimacy. Further, we found that perceived network size and perceived financial risk are two factors that prevent users from switching to expensive virtual gifts. In addition, information overload was introduced as a moderating variable. Our research model is shown in Figure 1.

### Hypothetical development

#### Immediacy

According to Wiener and Mehrabian (1968), immediacy allows users to perceive the urgency and importance of interacting through media platforms, such as users’ eagerness to use media platforms to view and respond to information. Immediacy has been shown to significantly influence users’ motivation and behavior in online environments, improving their immersion and engagement (Wang and Wu, 2019).For example, a study of student motivation showed some positive relationships between teacher immediacy and student engagement (Liu, 2021). Further, teachers can take full advantage of features such as emotional stickers, visual images, and audio provided by online course platforms to increase student engagement (Dixson et al., 2016).

On live streaming platforms, immediacy can greatly deepen users’ immersion and increase their engagement (Wang and Wu, 2019). A study investigating immediacy and real-time social interactions showed that the expectation of instant reciprocity when watching a live stream has a positive impact on the willingness to provide gifts (Deng and Chau, 2019).Currently, there are many users buying gifts on live streaming platforms, so streamers tend to prioritize interactions with users who buy expensive virtual gifts. This phenomenon leads to a kind of social competition, which may lead to anxiety about real-time interaction; this anxiety caused by social competition is called immediate interaction anxiety (Neil et al., 2012). Under the influence of immediate interaction anxiety, users who have purchased cheap virtual gifts are more likely to turn to buying expensive virtual gifts because the special effects of cheap virtual gifts are not as advanced as expensive virtual gifts, and even if they repeatedly purchase cheap gifts, it is difficult to attract significant attention from the streamer. Therefore, this study developed the following hypothesis:

*H1*: Immediate interaction anxiety positively influences users to switch from buying cheap virtual gifts to buying expensive virtual gifts.

#### Intimacy

Intimacy reflects an individual’s perceived level of closeness to or interaction with an exclusive partner, a perception that can be influenced not only by conversational exchanges but also by body language such as physical distance, eye contact, and smiling (Parker et al., 1978; Dixson et al., 2016; Woodcock and Johnson, 2019).

A direct way for streamers to create intimate scenarios is to express or imply the importance of intimacy through language to get users to buy virtual gifts (Wang, 2019). One study showed that the more emotional words or words with group meanings are used, the more relationships are fostered (Oh et al., 2020). For example, in some platforms, streamers will confess to users with nice voices and call them their husbands (wives) or brothers (sisters), giving users the illusion that the streamer is their emotional partner and inducing them to buy virtual gifts for them. Streamers also inform users that they can get more intimate privileges by purchasing more expensive virtual gifts, such as becoming an administrator of a streamer’s live streaming channel by purchasing 100 CNY (about USD 14.2) of virtual gifts, obtaining the private contact information (such as WeChat) of the streamer (100 CNY), and obtaining a private phone number of the streamer (200 CNY). This method of creating intimacy in terms of virtual intimacy aims to seduce users and lead them to buy expensive virtual gifts.

Nonverbal behaviors in digital platforms enhance the human perception of virtual intimacy, and the more nonverbal behaviors that are delivered, the higher the intimacy perceived by the user. This means that on digital platforms, nonverbal behaviors are what will cause virtual physical intimacy through body language. Some studies have confirmed that the more visual or physical contact in a live stream, the higher the intimacy generated (Woodcock and Johnson, 2019). Streamers often have good looks and sexy bodies, and if using these advantages to make a good intimate action can increase a user’s virtual physical intimacy, this may increase the user’s purchase intention (Zhang and Hjorth, 2017; Lee et al., 2019; Hou et al., 2020). However, as the number of users that watch a live broadcast increases, more and more users can buy virtual gifts for the streamer. As a streamer can only give a limited amount of intimacy, intimate interaction can only be provided to users who buy the most expensive virtual gifts, and at this time, because of social competition, users are more likely to buy expensive virtual gifts for the streamer. Therefore, the following hypotheses were developed:

*H2*: Verbal intimacy positively influences users to shift from buying cheap virtual gifts to buying expensive virtual gifts.

*H3*: Virtual physical intimacy positively influences users to shift from buying cheap virtual gifts to buying expensive virtual gifts.

#### Efficiency

Social presence theory states that immediacy can be measured using response efficiency, which suggests that when efficiency is low, immediacy will be low (Smedlund and Faghankhani, 2015). Therefore, in live streaming, when the interaction provided is inefficient, it is difficult for users to interact effectively with the streamer. Further, the reduction of interaction may reduce the chance of the streamer showing intimacy to the user. Therefore, this study analyzed the level of efficiency that hinders users’ willingness to purchase expensive gifts on live streaming platforms.

Perceived network size denotes an individual’s perception of the number of users using the same social network, and in general, the larger the network size, the more users tend to use it (Chang, 2018). However, a study also reported that users in live streaming rooms with fewer viewers are more likely to be engaged and motivated by the host than users in live streaming rooms with more people online, suggesting that the more users there are in a live streaming room, the less users tend to buy virtual gifts and the less they are interested in continuing to make purchases (Hilvert-Bruce et al., 2018). Further, a large number of users buy virtual gifts for the streamer, which also increases perceived affordability and ultimately reduces the payment behavior (Lee et al., 2019). In the context of this study, the number of users in a live streaming room is too high especially when too many users have already purchased expensive virtual gifts; when this occurs, users may feel that the immediate purchase of expensive virtual gifts does not necessarily increase the favorability of the streamer toward them and increase effective interaction, but rather brings a perceived burden. Therefore, this study developed the following hypothesis:

*H4*: Perceived network size negatively impacts users’ shift from buying cheap virtual gifts to buying expensive virtual gifts.

Financial risk is defined as the probability that a consumer will suffer a monetary loss as a result of a purchase if the product performs poorly or if the product is not worth buying (Featherman and Pavlou, 2003). Financial risk has been identified as a strong negative predictor of online purchase intentions (Kamalul Ariffin et al., 2018). In the context of this study, when users turn to buying expensive gifts when participating in live streaming, they may pay a significant financial cost. Some studies confirmed that in live streaming, the value of virtual gifts that users buy for streamers depends on the users’ financial affordability (Lee et al., 2019), and users may worry about whether buying expensive gifts will cause financial burden in their lives or whether buying expensive gifts will improve their interactions with streamers. These factors hinder users’ willingness to shift to buying expensive gifts. Therefore, this study developed the following hypothesis:

*H5*: Perceived financial risk negatively affects users’ shift from buying cheap virtual gifts to buying expensive virtual gifts.

#### Moderating role of information overload

Information overload can make users feel anxious and stressed and enter a state of information avoidance, making it difficult for them to use information effectively and make decisions based on limited information, which can easily lead to cognitive dissonance and wrong decisions (Krishen et al., 2011). When users are overloaded with information when watching live streaming, they may not be able to use the information comprehensively to judge the current level of relationship between them and the streamer, and it may be difficult for them to decide whether they should continue to interact or not with the streamer. At the same time, users experiencing information overload may avoid new information, and it may be difficult for them to feel the verbal intimacy and virtual physical intimacy constantly delivered by the streamer, making it likely that they will be less willing to switch to buying expensive virtual gifts. Therefore, this study developed the following hypotheses:

*H6*: Information overload negatively moderates the positive effect of immediate interaction anxiety on users’ willingness to shift to purchasing expensive virtual gifts.

*H7*: Information overload negatively moderates the positive effect of verbal intimacy on users’ willingness to shift to purchasing expensive virtual gifts.

*H8*: Information overload negatively moderates the positive effect of virtual physical intimacy on users’ willingness to shift to purchasing expensive virtual gifts.

One study pointed out that ambiguous situational perceptions can lead individuals to make incorrect purchases (Herjanto et al., 2021). From the user’s payment perspective, buying expensive virtual gifts in a live streaming platform can be considered as a negative shopping behavior, thus generating financial risks. Users may be cognitively dissonant in a state of information overload, and their perception of the situation may be blurred, so that they may become less aware of the perceived network size and financial risk, and thus engage in the negative behavior of purchasing expensive virtual gifts. From another perspective, research has confirmed that information overload may reduce users’ information comprehension, decrease their perceived behavioral control, and create the illusion of completing appropriate tasks without significant cost (Liu and Kuo, 2016). In the case of live streaming platforms, even if the network of streamer users and paying users is large, users may think that if they buy expensive virtual gifts, they will definitely promote effective interactions. With such a belief, they are likely to ignore their own affordability and disregard the financial risks associated with buying expensive virtual gifts. Therefore, this study developed the following hypothesis:

*H9*: Information overload moderates the effect of perceived network size on users’ willingness to shift to purchasing expensive virtual gifts.

*H10*: Information overload moderates the effect of perceived financial risk on users’ willingness to shift to purchasing expensive virtual gifts.

## Research methodology

### Sampling and data collection

This study used a questionnaire to collect data, and then tested the proposed hypotheses by analyzing these data. The study used a five-point Likert scale, with measures ranging from strongly disagree to strongly agree, to measure each construct. In order to improve the measurement items’ validity, the study slightly modified those proposed in the existing literature to fit the context of this study, and invited experts in the field to review the questionnaire. In addition, we conducted a small-scale pretest to assess the questionnaire’s reliability and validity. Details of the questionnaire can be found in Appendix A.

The questionnaire for this study was distributed online through social networks. Before distributing the questionnaire, the university’s scientific ethics committee was consulted, and the following measures were taken in order to ensure that there were no ethical issues in the questionnaire: (1) the questionnaire was made anonymous; (2) the content and purpose of the survey was clearly explained to the respondents; (3) all participants were free to answer or not answer the questionnaire; (4) the questionnaire did not involve personal or private information; (5) the data collected by the survey were only used for this study; (6) all participants were to receive an online gift upon completion of the questionnaire.

**Figure 2:**
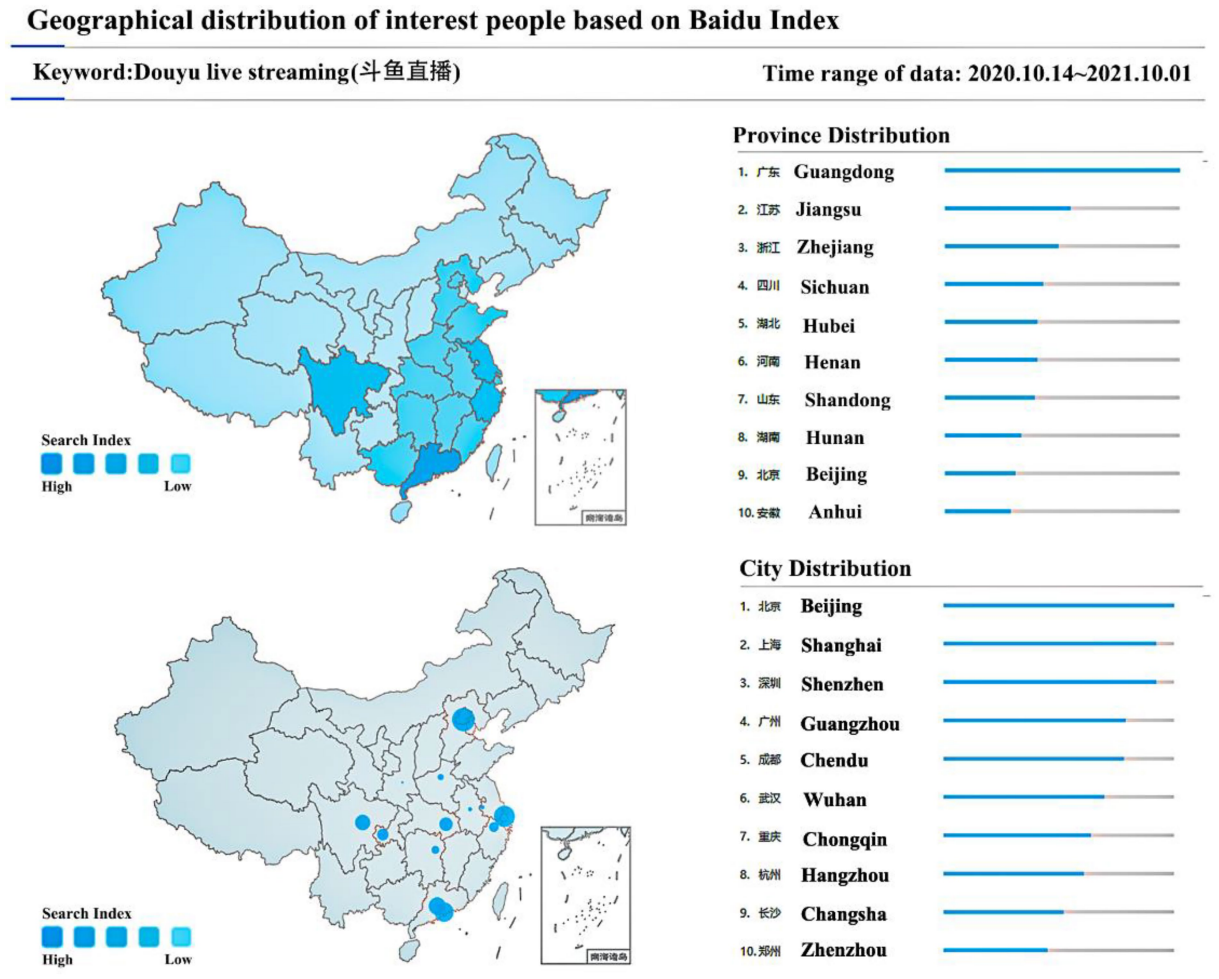
Level of attention paid to Douyu live streaming in China.

Since 2016, the number of live streaming platforms and users in China has exploded, having grown to over 100 live streaming platforms and over 300 million users. Further, the live streaming in China has grown from being worth USD 1.3 billion in 2016 and USD 6.4 billion in 2017 (Zhu et al., 2017), to close to USD 23.4 billion as of 2020. Douyu.com, the most famous Chinese online live streaming website, considered to be the Chinese version of Twitch (Zhang and Hjorth, 2017), attracted over 2 billion CNY (~USD 285 million) of investment in 2014, and has accumulated over 200 million registered users (Zhou et al., 2019). Therefore, users who use the Douyu live streaming platform were chosen as the target population for this study. According to the data for 2020–2021 provided in the Baidu Index^1^ (see Figure 2), in China, people living in Shenzhen, Guangdong province pay more attention to the Douyu live streaming platform than other people, so residents living in Shenzhen were chosen as the target survey population for the study.

One study suggested that virtual gifts over 100 CNY (about USD 14.2) on live streaming platforms are considered expensive gifts, and gifts over 500 CNY are considered luxury gifts (Zhang et al., 2019). Therefore, this study classified expensive virtual gifts as those over 100 CNY (about USD 14.2). To ensure the validity of the survey users, we set screening questions in our questionnaire, through which users who had not watched Douyu live, had not purchased expensive virtual gifts and had not shifted from buying cheap gifts to buying expensive gifts, were excluded from the survey.

This study used a snowballing approach to conduct the questionnaire survey. We randomly joined a chat group in Shenzhen using instant messaging software between 10 October 2021 and 25 October 2021, randomly recruited 100 respondents in the group who met the survey requirements, and asked each of them to invite as many friends living nearby to them as possible to participate in the survey; each valid respondent was given a reward of 7 CNY (about USD 1) for each invitation. Finally, a total of 513 responses were collected, and the data were filtered and deleted according to the survey requirements, resulting in 279 valid questionnaires (54.3%).

### Analysis methods

This study first used descriptive statistics to analyze the demographic characteristics of the obtained sample, and then tested the reliability, validity, and hypotheses in a structural equation model.

There are two types of structural equation models: a covariance-based structural equation model (CB-SEM) and a partial least squares based structural equation model (PLS-SEM). PLS-SEM was used in our study. Firstly, PLS-SEM is useful for prediction, and is less restrictive on sample sizes than other methods. Secondly, PLS-SEM is more suitable than CB-SEM for the measurement of complex models, especially for models larger than six variables (Hair et al., 2017). Thirdly, PLS-SEM allows for the analysis of non-normally distributed data (Hair et al., 2017). This study was an exploratory study with seven variables and data that did not conform to a normal distribution; therefore, the choice of PLS-SEM for the data analysis was more appropriate for this study.

## Empirical results

### Demographics

Demographic information was included in the survey. Of the respondents, 243 were male (87.1%) and only 36 were female (12.9%). The respondents were mainly between 21 and 30 years old (*N* = 155, 55.6%). Their education levels were mainly the bachelor’s degree (*N* = 95, 34.1%) and junior college degree levels (*N* = 81, 29%). The respondents mainly watched female live streaming in the facial attractive section of the live streaming platform (*N* = 263, 94.2%), while a few watched male online streamers in the facial attractive section (*N* = 16, 5.7%). There were 72.8% people (*n* = 203) who mainly bought high-value virtual gifts using cash, 24.4% people (*n* = 68) who chose credit cards and other credit payment methods to buy high-value virtual gifts, and 2.9% people (*n* = 8) who bought high-value virtual gifts through peer-to-peer lending. Of the respondents, 19.4% interviewees (*n* = 54) had a monthly income of less than CNY 2,000 (USD 285), 23.7% had a monthly income of between CNY 2,000 and CNY 4,000 (USD 285–USD 571), 20.8% had a monthly income of between CNY 4,000 and CNY 6,000 (USD 571–USD 857), while 20.4% had a monthly income of between CNY 6,000 and CNY 8,000 (USD 857–USD 1,142). In addition, 15.8% (*n* = 44) of respondents earned more than CNY 8,000 (USD 1,142); of the respondents, 24.4% said that they had bought virtual gifts worth more than CNY 1,000 (USD 142), 35.8% said they had bought gifts worth between CNY 500 and CNY 1,000 (USD 71–USD 142), while 39.8% said that they had bought gifts worth between CNY 100 and CNY 500 (USD 14.2–USD 71). There were 233 respondents (84.4%) who were of the opposite sex to the online streamers they watched, among whom 82.4% were males (*n* = 230) watching female online streamers, and 1% were females (*n* = 3) watching male online streamers. There were 46 respondents (16.5%) who usually watched facial attractive online streamers of the same gender as themselves; among these, 13 were male (4%) and 33 female (11.8%).

To assess non-response bias in the sample, a paired t-test was performed on the demographic items of the top 20 respondents and the bottom 20 respondents, which showed no significant difference.

**Figure 3:**
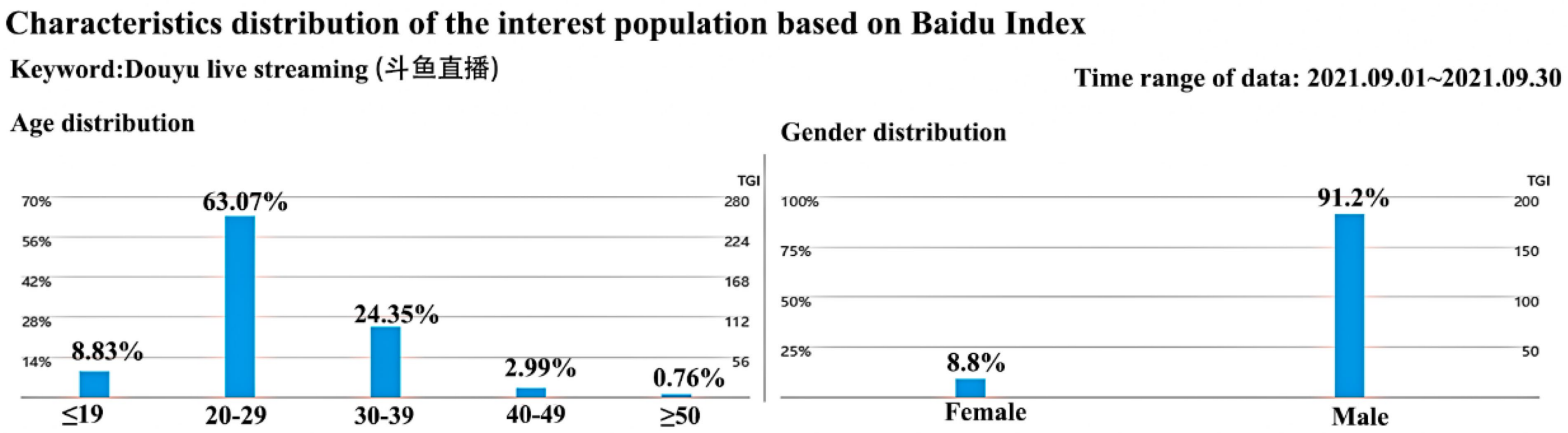
Gender and age distribution of Douyu live streaming in the Baidu Index.

As pointed out, most of the respondents were male, concentrated between the ages of 21 and 30 years; we did not consider this as having an effect on the representativeness of the research results. As Figure 3 shows, which is based on the entire Baidu index network for statistical data, the gender distribution of China’s internet-watching crowd is such that males account for 91.2%, while females account for 8.8%. Additionally, age is mainly concentrated between 20 and 29 years, which is similar to the age profile of our respondents. Furthermore, our study measured the effects of the control variables on the endogenous variables, none of which had a significant effect.

### Common method bias

Common method bias is a problem that tends to occur in questionnaires. Harman’s single factor analysis (HFA) is widely used to estimate common method bias in social science research (Harman, 1976). Podsakoff et al. (2003) suggested that a single factor can be extracted, and that if the variance of this factor is less than 40%, it means that the survey data are less affected by common method bias. The variance of the extracted single variable in this study was 23.62% (<40%).

Further, we performed a common method bias test using the PLS-SEM recommended approach. Kock (2015) proposed that the occurrence of Full-VIFs values > 3.3 is considered an indication of pathological covariance, meaning that a given model may have been affected by common method bias. Therefore, if all Full-VIFs are equal to or below 3.3, the model can be considered free of common method bias. In the present study, the Full-VIFs value for each structure was below 3.3. Therefore, based on the results of the tests for both methods on common method bias, it can be considered that common method bias was not an issue in this study.

### Measurement model

In this study, we first assessed the model quality, and the results are shown in Table 2. First, we performed a collinearity diagnosis, and the VIF between each aspect was less than 10, indicating that there was no collinearity problem in this study. Then, we used composite reliability to assess the internal consistency reliability, and as shown in Table 2, the CR of each construct was higher than 0.7, and the Cronbach α was higher than 0.7, indicating that the scale used in each dimension was highly reliable (Hair et al., 2017). This study determined the convergent validity by assessing the average variance extracted(AVE). When the AVE value is greater than 0.5, the criterion of convergent validity is proven to be satisfied. In this study’s model, the AVEs were all higher than 0.5, indicating that the scale had good convergent validity (Hair et al., 2017).

**Table 2:** Results of confirmatory factor analysis (CFA) for the measurement model.

Latent variable	Item	Loading	Cronbach’s a	CR	AVE	VIF
II	II1	0.922	0.846	0.906	0.762	1.192
	II2	0.813				
	II3	0.881				
VI	VI1	0.934	0.856	0.909	0.769	1.031
	VI2	0.788				
	VI3	0.903				
PI	PI1	0.920	0.837	0.902	0.755	1.029
	PI2	0.832				
	PI3	0.852				
NS	NS1	0.848	0.859	0.914	0.779	1.164
	NS2	0.874				
	NS3	0.924				
FR	FR1	0.935	0.852	0.909	0.769	1.196
	FR2	0.802				
	FR3	0.888				
IO	IO1	0.928	0.804	0.882	0.714	1.351
	IO2	0.761				
	IO3	0.839				
SI	SI1	0.881	0.708	0.836	0.631	-
	SI2	0.757				
	SI3	0.737				
PB	PB1	0.882	0.767	0.866	0.684	-
	PB2	0.774				
	PB3	0.820				

II, Immediate interaction anxiety; VI, verbal intimacy; PI, virtual physical intimacy; NS, perceived network size; FR, perceived financial risk; IO, information overload; SI, switching intention; PB, Purchase behavior.

Discriminant validity is first evaluated by measuring the square root of AVE. The square root of AVE for each structure should be greater than the correlation between the structure and all other constructions, indicating that the evaluation of convergence validity is correct (Fornell and Larcker, 1981). In this study, the square root of each surface was greater than all the correlation coefficients (see Table 3). Next, the factor loading needed to be tested. The loading on all items in this study was higher than 0.7(Fornell and Larcker, 1981). This study also adopted the heterotrait–monotrait ratio (HTMT) method. The results are shown in Table 4. The HTMT values between variables met the requirement of being less than 0.85. These results show that the discriminant validity of the scale met the requirements of the study (Hair et al., 2017).

**Table 3:** Factor correlation coefficients and square roots of the AVE.

	II	VI	PI	NS	FR	SI	PB	IO
II	0.873							
VI	−0.026	0.877						
PI	−0.026	−0.09	0.869					
NS	−0.25	−0.005	−0.025	0.883				
FR	−0.24	0.083	−0.028	0.244	0.877			
SI	0.267	0.181	0.29	−0.344	−0.299	0.795		
PB	0.181	0.111	0.043	−0.214	−0.153	0.412	0.827	
IO	−0.352	−0.07	−0.114	0.324	0.352	−0.54	−0.289	0.845

II, Immediate interaction anxiety; VI, verbal intimacy; PI, virtual physical intimacy; NS, perceived network size; FR, perceived financial risk; IO, information overload; SI, switching intention; PB, Purchase behavior.

**Table 4:** Heterotrait–monotrait ratio (HTMT) test.

	II	VI	PI	NS	FR	SI	PB	IO
II								
VI	0.053							
PI	0.049	0.111						
NS	0.296	0.066	0.035					
FR	0.271	0.115	0.086	0.287				
SI	0.332	0.220	0.365	0.425	0.363			
PB	0.231	0.129	0.060	0.262	0.189	0.561		
IO	0.417	0.075	0.122	0.364	0.408	0.680	0.364	

### Structural model

After ensuring the reliability and validity of the model, we measured the structural model to test the research hypothesis, and the results are shown in Table 5. Among the factors influencing users to switch to purchasing expensive virtual gifts, immediate interaction anxiety had a significant positive effect on users’ switching intentions (ß = 0.172, *p* < 0.05), and therefore H1 was supported. Verbal intimacy had a significant positive effect on users’ switching intentions (ß = 0.229, *p* < 0.001), and therefore, H2 was supported. Virtual physical intimacy had a significant positive effect on users’ switching intention (ß = 0.308, p < 0.001), and therefore H3 was also supported. Perceived network size had a significant negative effect on users’ switching intentions (ß = −0.244, *p* < 0.001), and therefore H4 was supported. Perceived financial risk also had a significant negative effect on users’ intention to switch (ß = −0.213, *p* < 0.001), and therefore H5 was supported. Switching intention had a positive effect on users’ purchase behavior (ß = 0.410, *p* < 0.001), and therefore H6 was supported.

**Table 5:** Hypothesis testing results.

Hypotheses	ß	STDEV	T statistics	*P-*values	Result
H1: II → SI	0.172	0.050	3.442	0.001	Support
H2: VI → SI	0.229	0.054	4.272	0.000	Support
H3: PI → SI	0.308	0.049	6.313	0.000	Support
H4: NS → SI	−0.244	0.053	4.636	0.000	Support
H5: FR → SI	−0.213	0.052	4.130	0.000	Support
H6: SI → PB	0.410	0.059	6.902	0.000	Support

II, Immediate interaction anxiety; VI, verbal intimacy; PI, virtual physical intimacy; NS, perceived network size; FR, perceived financial risk; IO, information overload; SI, switching intention; PB, Purchase behavior.

This study also examined the goodness of fit (GOF) of the model. In PLS-SEM, an SRMR value below 0.08 indicates that the model has an acceptable goodness of fit. An SRMR value of 0.061 was obtained for the model in this study, indicating that the model had an acceptable goodness of fit (Fornell and Larcker, 1981).

### Moderating effects analysis

This study measured the moderating effect in two steps. In the first step, the degree of significance of the moderating effect was measured. In the second step, the strength of the moderating effect was measured by calculating the F^2^ in the significant effect. F^2^ is calculated as (R^2^interaction mod − R^2^main effects model)/(1 − R^2^main effects model). If F^2^ is between 0.02 and 0.15, a small moderating effect is indicated, if it is between 0.15 and 0.35, the moderating effect is medium, and if it is greater than 0.35, the moderating effect is high (Chin, 1998).

The calculated results are shown in Table 6. Information overload moderately and significantly moderated the positive effect of immediate interaction anxiety on users’ willingness to switch, and therefore, H7 was supported (ß = −0.159, *p* < 0.05). Information overload did not have a significant moderating effect on the relationship between verbal intimacy and users’ willingness to switch (ß = −0.092, n.s.), and therefore H8 was not supported. There was no significant moderating effect of information overload in the relationship between virtual physical intimacy and users’ willingness to switch (ß = −0.048, n.s.), and therefore H9 was not supported. Information overload moderately and significantly moderated the negative effect of perceived network size on users’ willingness to switch (ß = 0.121, *p* < 0.05.), and H10 was therefore supported. Information overload also moderately and significantly moderated the negative effect of perceived financial risk on users’ willingness to switch (ß = 0.173, *p* < 0.05.), and therefore H11 was also supported.

**Table 6:** Moderating effect analysis results.

Path	ß	STDEV	T statistics	*P*-values	R^2^ (interaction)	R^2^ (main effects)	F^**2**^	Result
H7: II*IO → SI	−0.159	0.063	2.517	0.012	0.447	0.328	0.177	Support
H8:VI*IO → SI	−0.092	0.067	1.377	0.168	0.433	0.328	−	Reject
H9: PI*IO → SI	−0.048	0.062	0.781	0.435	0.427	0.328	−	Reject
H10: NS*IO → SI	0.121	0.061	1.973	0.049	0.439	0.328	0.165	Support
H11:FR*IO → SI	0.173	0.059	2.914	0.004	0.451	0.328	0.183	Support

II, Immediate interaction anxiety; VI, verbal intimacy; PI, virtual physical intimacy; NS, perceived network size; FR, perceived financial risk; IO, information overload; SI, switching intention.

**Figure 4:**
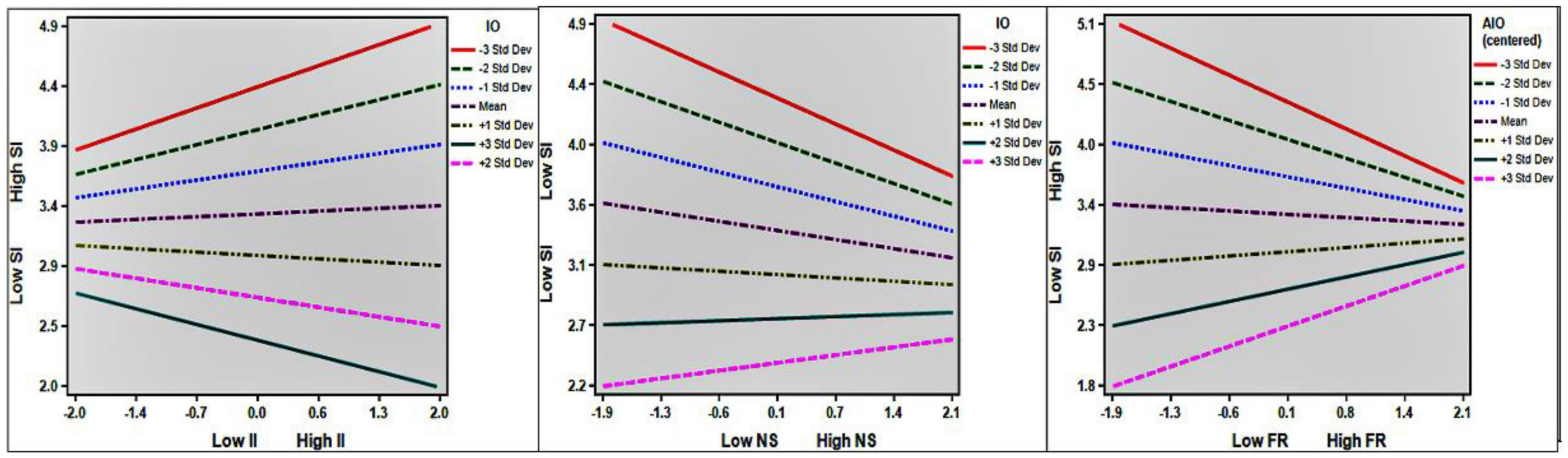
Moderation effect slopes II, Immediate interaction anxiety; NS, perceived network size; FR, perceived financial risk; IO, information overload; SI, switching intention..

According to the slope diagrams shown in Figure 4, it can be seen that as the degree of information overload increased, the positive effect of immediate interaction anxiety on willingness to switch gradually decreased, the negative effect of perceived network size on users’ willingness to switch gradually decreased, and the negative effect of perceived financial risk on users’ willingness to switch gradually decreased.

## Discussion and conclusion

### Key findings

The first aim of this study was to analyze, based on the perspective of social presence theory, the factors that influence live streaming users to switch from buying cheap virtual gifts to buying expensive virtual gifts. First, immediate interaction anxiety positively influences users to shift from buying cheap virtual gifts to buying expensive virtual gifts. Streamers make users perceive the urgency and importance of interaction through live streaming platforms, thus influencing users’ consumption motivation, while immediate interaction with streamers can make users feel excited (Wang and Wu, 2019). Users expect to interact consistently and frequently with their favorite streamers, and this expectation has a positive impact on their intention to purchase virtual gifts (Deng and Chau, 2019). However, the existence of social competition means that not all users are able to interact with their favorite streamers in real time. The more people want to stand out from their social competition, the more anxious they become (Neil et al., 2012). Therefore, the most direct solution to anxiety may be to turn to the consumption of expensive virtual gifts.

Second, the verbal intimacy of the streamer influences users to purchase expensive virtual gifts. One direct way that streamers create intimate situations is by building intimacy through language (Wang, 2019). Users are easily tempted by streamers to give expensive virtual gifts (Lee et al., 2019), for example, streamers call users their husbands (wives) or brothers (sisters) and express the wish that they send them expensive virtual gifts. Streamers also inform users that giving expensive virtual gifts can lead to more intimate privileges, such as the streamer’s personal contact information (Zhang et al., 2019).

Third, the virtual physical intimacy that streamers confer on users is also a factor that causes users to switch from buying cheap gifts to buying expensive virtual gifts. The higher the virtual physical intimacy in live streaming, the greater the intimacy’s impact (Woodcock and Johnson, 2019). Making good use of intimate gestures can increase users’ virtual physical intimacy and their consumption intention (Lee et al., 2019; Hou et al., 2020), as most streamers convey visual intimacy by smiling or showing their body shape. However, at present, there are still some loopholes in the management of live streaming platforms. For example, some streamers have added sexually suggestive actions to increase the virtual physical intimacy of users (Zhang and Hjorth, 2017; Lee et al., 2019). In addition, social competition makes the number of users who receive this virtual physical intimacy limited, making users consider buying expensive gifts (Zhou et al., 2019).

Fourth, in online live streaming, the number of real-time participants is one of the factors that prevents users from buying expensive virtual gifts. Generally speaking, the larger the platform size, the more likely users are to use it (Pal et al., 2020). However, in online live streaming, this association is reversed (Hilvert-Bruce et al., 2018). The smaller the number of users in live streaming, the more likely users are to get the opportunity to interact with the streamer and thus gain intimacy from the streamer. Therefore, the larger the number of users in live streaming, the less users are willing to buy expensive virtual gifts.

Fifth, perceived financial risk is also an important factor that discourages users from purchasing expensive virtual gifts. Financial risk is a strong negative factor for online live streaming users’ purchase intention (Kamalul Ariffin et al., 2018). In live streaming, the value of virtual gifts purchased by the user for the streamer depends on the user’s financial affordability (Lee et al., 2019). Users will consider whether buying expensive virtual gifts will place a financial burden on their lives and whether such a purchase will improve their intimacy with the streamer.

Another objective of this study was to clarify the moderating role of information overload in online live streaming in users’ shift from buying cheap virtual gifts to buying expensive virtual gifts. Firstly, information overload in live streaming reduces users’ immediate interaction anxiety, and predicts their ability to purchase high-value virtual gifts. Information overload is thought to cause user fatigue (Zhang et al., 2016). Users’ becoming fatigued certainly reduces their need for immediate interaction with the streamer, and thus does not lead them to the purchase of expensive virtual gifts. Information overload can also lead to cognitive impairment and decision errors (Hu and Krishen, 2019), which may be reflected in users’ misjudgment of their relationship with the streamer during live streaming. Users may believe that they have achieved their goals and thus may not purchase expensive virtual gifts.

Secondly, information overload in live streaming slows down the role of perceived network size and financial risk in preventing users from purchasing expensive virtual gifts. The most important point about information overload is that it leads to cognitive dissonance in and the making of wrong decisions by individuals (Cao et al., 2021). After experiencing information overload, users avoid information (Farooq et al., 2021), and may even enter a state of information room bubble (Hayat et al., 2019). That is, when users are caught up in too many temptations from streamers, they are unable to fully analyze the external (network size) and internal (financial risk) environments to make a rational decision about whether to buy an expensive virtual gift. Streamers may keep sending tempting signals while the users are in a backroom bubble, where they only hear information that prompts them to buy expensive virtual gifts.

In this study, information overload was not confirmed to have a moderating effect on verbal intimacy, virtual physical intimacy, and intention to purchase expensive virtual gifts. A possible explanation for this is that most of the streamer’s information is intended to increase intimacy with the user. Such messages can lead to two effects. When too much intimacy information leads to information overload due to reciprocal psychological expectations and real-time interactions, users’ intention to purchase expensive virtual gifts becomes urgent. However, such information is also greatly valued by the user, but too much of this can cause social overload, leaving the user to feel unsatisfied and powerless (Raza et al., 2020). Thus, the moderating effect of information overload on this relationship may become less pronounced.

### Theoretical contributions

This study provides several contributions to the literature. First, while many studies have focused on the impact of social presence on live streaming participation and consumption (Li et al., 2018; Liu et al., 2020), this study looked at the core concept of social presence and analyzed in more detail how the core subconcepts of social presence affect users’ willingness to purchase expensive virtual gifts.

Second, nonverbal communication in the original social presence theory is considered a subconcept derived from intimacy (Parker et al., 1978). This study further developed and integrated the study of nonverbal communication and intimacy in the live streaming environment, extending them to verbal intimacy and virtual physical intimacy, which helps to better explain the impact of intimacy on users’ shift to expensive consumption in live streaming, enriching the social presence theory and extending the scope of its use.

Third, efficiency in the original social presence theory is considered a derivative sense of immediacy (Smedlund and Faghankhani, 2015). This study separated efficiency in the context of live streaming and extended it to perceived network size and perceived financial risk, analyzing the hindering effects of both on users’ expensive consumption intentions in live streaming, enriching the social presence theory while extending the use of the theory.

Finally, a large number of studies have pointed out that information overload, as a source of stimuli, has a direct impact on social platform users’ behavior (Cao and Sun, 2018). The present study introduced information overload factors as moderating variables in the analysis of users’ willingness to spend on social platforms, especially the moderating role of information overload in influencing users’ willingness to engage in such spending, which enriches the use of information overload theory and contributes to the theoretical study of social commerce marketing.

### Practical contributions

This study also has practical contributions that are instructive for the marketing of live streaming and that can be extended to a wider range of social commerce activities.

This study argues that for practitioners of live streaming, they need to control the level of interaction given to users and the intimacy conveyed according to the consumption situation. For low spending users, it can be necessary to give them some interaction and intimacy at the time of low spending, to the extent that they happen to feel the streamers’ presence, and directly ask them to buy more expensive virtual gifts after introducing them to the benefits of buying these. Then, if they are not moved, it is advised that they quickly reduce the amount of interaction with these users, and show them the intimacy and interaction they provide to high spending users, tempting the former to buy more expensive virtual gifts. For users who have already purchased expensive virtual gifts, it is fine to maintain a normal level of interaction and intimacy, and not to directly request this segment of users to continue spending too much, but rather to convey virtual physical intimacy to them through physical actions within the allowed range. The streamer should have a certain sense of a given user’s financial ability through their daily interaction and consumption habits. For users who are not financially capable but are eager to improve the amount of interaction they receive, streamers can engage in excessive interaction with such users during downturns in the number of people in their live rooms, such as passing on a sense of intimacy that these users do not normally enjoy, giving them an overload of virtual emotions and virtual physical intimacy to stimulate them to purchase expensive gifts. In addition, if the streamer has a large number of people in their live room and many people buying expensive gifts, it is necessary to attract some new users who are willing to buy expensive virtual gifts, although it is not necessary to worry about income at this time. For example, a streamer can choose to give special intimacy to users who have been spending cheaply but have some potential to spend in greater amounts when there are a lot of people in their live room. Note that here the emphasis is on giving special intimacy rather than excessive interaction, so that such users experience intimacy overload and reduce their perception of user size and thereby turn to buying expensive virtual gifts.

This study suggests that for live streaming users, the most important thing is to improve their resistance to the temptation of streamers and prevent themselves from engaging in impulsive spending behaviors due to information overload. For example, users can choose some famous streamers, which often have very large user followings and high incomes, which can help users with having accurate judgment of their ability to pay and reduce their willingness to buy expensive virtual gifts. Financial factors are the most fundamental factors leading to expensive spending, so for users who are weak in resisting the temptation of streamers, it is recommended to set a spending limit or eliminate the credit payment means. Finally, users who are addicted to consuming in live streaming can try to put down their phones and fill their leisure time with brand new hobbies, such as learning new knowledge and skills, arranging sports and fitness activities, minimizing the time and frequency of using computers and smartphones, reducing the use of live streaming APPs, and devoting limited time to things that are more beneficial to physical and mental development.

### Limitations and future directions

This study has some shortcomings. First, information overload can be divided into several types: for example, with the development of live streaming on the Internet, live streaming platforms are becoming more and more functional, and the acquisition of expensive virtual gifts takes place not only through direct purchases, but also through the emergence of gamified acquisition methods such as lotteries. The visual effects provided by platforms after expensive gifts are given are also becoming more complex, and these point directly to the possibility of causing system feature overload, which was not analyzed in this study. It is necessary, then, to analyze in subsequent studies the impact of different types of information overload on users’ shift to buying expensive virtual gifts. Second, new and experienced users differ in their willingness to purchase expensive gifts, and these types of users were not distinguished between in this study; this therefore needs to be analyzed in a follow-up study. Third, this study was conducted in China, and in particular, in Shenzhen, Guangdong province, which is one of the more economically developed regions in China, which implies that users’ switching intentions may be different in less economically developed regions, and means further that the switching intentions of users in other countries remain unclear and therefore need to be further studied.

## Data availability statement

The data presented in this study are available upon request from the corresponding author. The data are not publicly available for ethical reasons.

## Author contributions

JC: conceptualization, methodology, investigation, software, formal analysis, data curation, and writing—original draft. DL: conceptualization, writing—review and editing, and supervision. MS and GZ: investigation and writing—review and editing. All authors contributed to the article and approved the submitted version.

## Conflict of interest

The authors declare that the research was conducted in the absence of any commercial or financial relationships that could be construed as a potential conflict of interest.

## Publisher’s note

All claims expressed in this article are solely those of the authors and do not necessarily represent those of their affiliated organizations, or those of the publisher, the editors and the reviewers. Any product that may be evaluated in this article, or claim that may be made by its manufacturer, is not guaranteed or endorsed by the publisher.

## Supplementary material

The Supplementary material for this article can be found online at: https://www.frontiersin.org/articles/10.3389/fpsyg.2022.997651/full#supplementary-material

## Footnotes

^1^ http://index.baidu.com
