# Supplementary material for: Influencing factors of users’ shift to buying expensive virtual gifts in live streaming: Empirical evidence from China
Source: Front Psychol. 2022 Nov 30;13:997651. doi: 10.3389/fpsyg.2022.997651 (PMC9748564; doi:10.3389/fpsyg.2022.997651)
Supplement: Supplementary file 1 [file Table_1.docx]

## Appendix A

| **Factors** | **Serial Num.** | **Item** | **Reference** |
| --- | --- | --- | --- |
| Immediate interaction anxiety  (II) | II1 | After buying small gifts, it didn't improve my interaction with the streamer, which made me nervous. | Hwang, et al. [82] |
|  | II2 | After buying small gifts, the real-time interaction between me and the streamer did not meet my expectations. |  |
|  | II3 | After buying small gifts, I can't accept the reality that I can't interact with the streamer in real time. |  |
| Verbal intimacy  (VI) | VI1 | Online streamer has confirmed my idea and emotion through conversation. | Sinclair and Dowdy [83] |
|  | VI2 | Online streamer shares me her internal idea and feeling through conversation. |  |
|  | VI3 | Online streamer takes care of me and cares about me through conversation. |  |
| Virtual physical intimacy  (PI) | PI1 | Online streamer’ behavior make me feel like they have touched my body (like kiss, hug etc.) | Wiederman [84] |
|  | PI2 | Online streamer has shown me they hot body through some actions. |  |
|  | PI3 | I have felt sexual cue in some actions. |  |
| Perceived  network size  (NS) | NE1 | Many people like watching the online streamer who I usually see. | Chang [63] |
|  | NE2 | The online streamer I often see owns a lot of audience every time when they are in live streaming channel. |  |
|  | NE3 | In my social network, there are many people viewing the same online streamer who I often see. | Pal, Herath, De and Rao [75] |
| Perceived  financial risk  (FR) | FB1 | High value virtual is not worth spending my money on it. | Kamalul Ariffin, Mohan and Goh [65] |
|  | FB1 | Purchase high value virtual gift is money-wasting behavior. |  |
|  | FB2 | I will not overspend for high value virtual gift. |  |
| Information Overload  (IO) | SIO1 | Online streamer has conveyed so much of various information that I missed the information nearly related to myself | Hwang, Hong, Tai, Chen and Gouldthorp [82] |
|  | SIO2 | Online streamer has conveyed too much information. |  |
|  | SIO3 | I have no idea of the numerous information the online streamer conveys to me | Karr-Wisniewski and Lu [85] |
| Switching intention  (SI) | SI1 | After buying small-value virtual gifts, I will try to buy high-value virtual gift for online streamer. | Chang, et al. [86] |
|  | SI2 | After buying small-value virtual gifts, the possibility I purchase high-value virtual gift for online streamer is high. |  |
|  | SI3 | After buying small-value virtual gifts，I plan to buy high-value virtual gift for online streamer. | Tang and Chen [87] |
| Purchase  behavior  (PB) | PB1 | After buying small virtual gifts, I bought large virtual gifts. | Lin, et al. [88] |
|  | PB2 | After buying small virtual gifts, I buy large virtual gifts more often. |  |
|  | PB3 | After buying small virtual gifts, I am very happy to buy large virtual gifts on the live streaming platforms. |  |
